# The Yin and Yang of Microglia-Derived Extracellular Vesicles in CNS Injury and Diseases

**DOI:** 10.3390/cells13221834

**Published:** 2024-11-06

**Authors:** Mousumi Ghosh, Damien D. Pearse

**Affiliations:** 1 The Miami Project to Cure Paralysis, University of Miami Miller School of Medicine, Miami, FL 33136, USA; dpearse@med.miami.edu; 2 The Department of Neurological Surgery, University of Miami Miller School of Medicine, Miami, FL 33136, USA; 3 Department of Veterans Affairs, Veterans Affairs Medical Center, Miami, FL 33136, USA; 4 The Neuroscience Program, University of Miami Miller School of Medicine, Miami, FL 33136, USA; 5 The Interdisciplinary Stem Cell Institute, University of Miami Miller School of Medicine, Miami, FL 33136, USA

**Keywords:** microglia, exosome, extracellular vesicles, biogenesis, uptake, inflammation, neurodegeneration, neuroprotection, neurorepair, CNS injury

## Abstract

Microglia, the resident immune cells of the central nervous system (CNS), play a crucial role in maintaining neural homeostasis but can also contribute to disease and injury when this state is disrupted or conversely play a pivotal role in neurorepair. One way that microglia exert their effects is through the secretion of small vesicles, microglia-derived exosomes (MGEVs). Exosomes facilitate intercellular communication through transported cargoes of proteins, lipids, RNA, and other bioactive molecules that can alter the behavior of the cells that internalize them. Under normal physiological conditions, MGEVs are essential to homeostasis, whereas the dysregulation of their production and/or alterations in their cargoes have been implicated in the pathogenesis of numerous neurodegenerative diseases, including Alzheimer’s disease (AD), Parkinson’s disease (PD), multiple sclerosis (MS), spinal cord injury (SCI), and traumatic brain injury (TBI). In contrast, MGEVs may also offer therapeutic potential by reversing inflammation or being amenable to engineering for the delivery of beneficial biologics or drugs. The effects of MGEVs are determined by the phenotypic state of the parent microglia. Exosomes from anti-inflammatory or pro-regenerative microglia support neurorepair and cell survival by delivering neurotrophic factors, anti-inflammatory mediators, and molecular chaperones. Further, MGEVs can also deliver components like mitochondrial DNA (mtDNA) and proteins to damaged neurons to enhance cellular metabolism and resilience. MGEVs derived from pro-inflammatory microglia can have detrimental effects on neural health. Their cargo often contains pro-inflammatory cytokines, molecules involved in oxidative stress, and neurotoxic proteins, which can exacerbate neuroinflammation, contribute to neuronal damage, and impair synaptic function, hindering neurorepair processes. The role of MGEVs in neurodegeneration and injury—whether beneficial or harmful—largely depends on how they modulate inflammation through the pro- and anti-inflammatory factors in their cargo, including cytokines and microRNAs. In addition, through the propagation of pathological proteins, such as amyloid-beta and alpha-synuclein, MGEVs can also contribute to disease progression in disorders such as AD and PD, or by the transfer of apoptotic or necrotic factors, they can induce neuron toxicity or trigger glial scarring during neurological injury. In this review, we have provided a comprehensive and up-to-date understanding of the molecular mechanisms underlying the multifaceted role of MGEVs in neurological injury and disease. In particular, the role that specific exosome cargoes play in various pathological conditions, either in disease progression or recovery, will be discussed. The therapeutic potential of MGEVs has been highlighted including potential engineering methodologies that have been employed to alter their cargoes or cell-selective targeting. Understanding the factors that influence the balance between beneficial and detrimental exosome signaling in the CNS is crucial for developing new therapeutic strategies for neurodegenerative diseases and neurotrauma.

## 1. Introduction

Microglia are the primary innate immune cells of the central nervous system (CNS), essential to the maintenance of neural homeostasis and the body’s response to pathological challenges [1,2,3]. Microglia are involved in the brain’s immune defense, providing constant surveillance by orchestrating an inflammatory response to infection or disease. Microglia are also involved in neurotrophic support and play a key role in synaptic remodeling including synaptic pruning, the phagocytosis of debris, and cytokine secretion [2,3]. The unique characteristics and functional roles of microglial subtypes that arise from the cell’s highly plastic and diverse nature have been examined within both healthy and diseased states [4]. Recent advancements have identified a novel mechanism by which microglia influence the intricate processes of cellular communication, the release of extracellular vesicles (EVs). EVs are defined as a heterogeneous family of cell-derived membrane-bound vesicles, typically categorized into three types based on their size, origin, and how they are generated: exosomes (~30–150 nm diameter in size and released via multivesicular bodies), microvesicles (~0.1–1.0 μm diameter and produced from the budding of the plasma membrane), and apoptotic bodies (~1–5 μm diameter, evolving as a result of apoptosis) [5,6,7,8]. Recent studies have uncovered additional types of nanosized EVs including ectosomes, exomeres, and supermeres [9,10]. From a functional standpoint, EVs mediate intercellular communication by transferring proteins, lipids, and nucleic acids between cells [11,12,13,14,15] and have been implicated in numerous physiological and pathological processes [16,17,18,19].

**Figure 1:**
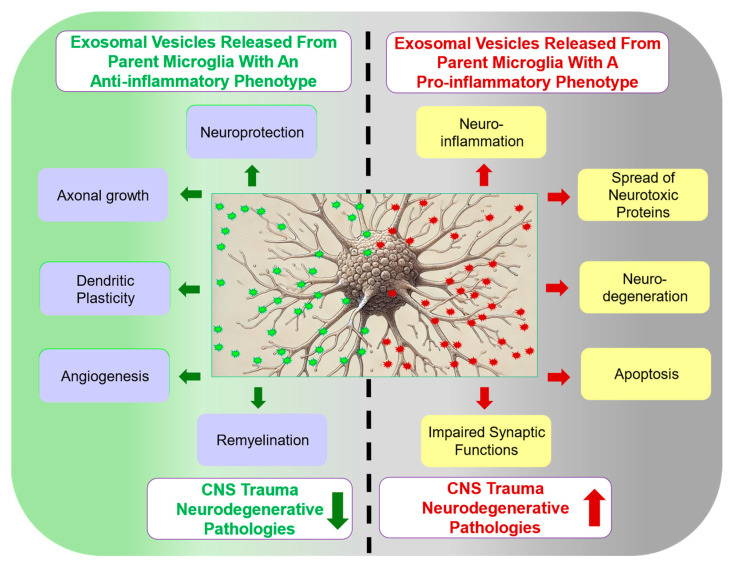
The function of microglial exosomes is influenced by the phenotypic state of the parent cell. Exosomal vesicles released from anti-inflammatory microglia are depicted in green, representing their neuroprotective and pro-regenerative roles (indicated by green arrows). In contrast, EVs derived from pro-inflammatory microglia are shown in red, indicating their association with heightened neuroinflammation, cell death and neurodegenerative conditions (indicated by red arrows).

Microglial exosomes (MGEVs) play a crucial role in regulating the adult neural stem niche [20,21] and contribute to normal brain function by facilitating neuron–glia communication [22]. On the other hand, they have been shown to exacerbate various neurodegenerative conditions such as Alzheimer’s disease (AD) [23,24], Parkinson’s disease (PD) [25], and amyotrophic lateral sclerosis (ALS) [26] via the intercellular transmission of misfolded and nonfunctional proteins, as well as inducing pro-inflammatory degenerative responses [16,27,28]. Conversely, anti-inflammatory MGEVs play a key role in promoting neuroprotection and repair. Their cargo typically includes anti-inflammatory mediators, neurotrophic factors, and molecular chaperones, which help to reduce inflammation, support neuronal survival, and enhance synaptic plasticity, fostering a more favorable environment for neural recovery. The objective of this review is to explore the dual nature of MGEVs in the context of neurotrauma and neurodegenerative diseases. In particular, MGEV characteristics, release patterns, pro-proliferative and pro-apoptotic effects on neurons and other glial cells, immunomodulatory effects, and regulation of the extracellular microenvironment will be discussed [29]. By examining both the beneficial and deleterious effects of MGEVs (Figure 1), we aim to discern important points of conjuncture in these processes that could improve the development of therapeutic interventions employing the delivery or antagonism of MGEVs in CNS injury and disease.

## 2. The Biology of Microglia Exosomes

### 2.1. Biogenesis

**Figure 2:**
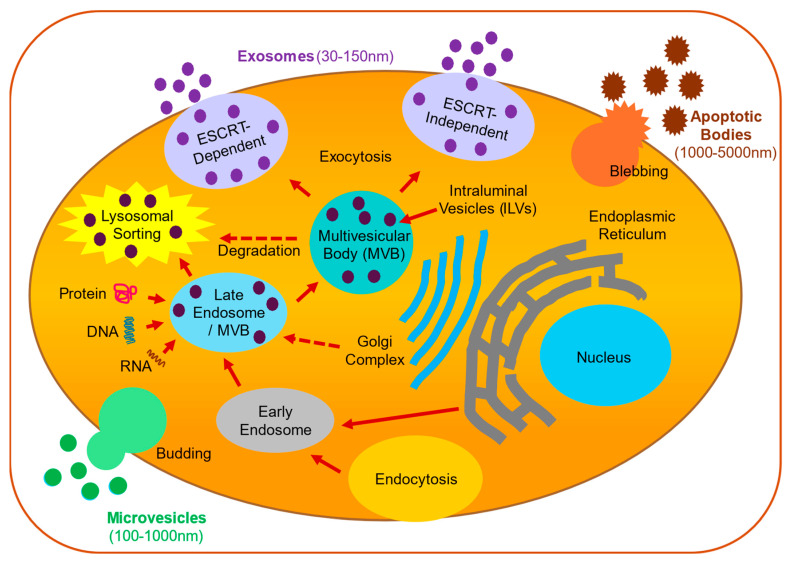
Biogenesis of exosomal vesicles.

The mechanisms of exosome biogenesis remain poorly understood. The current consensus in the scientific literature is that EVs originate either from intracellular endocytic trafficking or the plasma membrane [30,31] (Figure 2). The main characteristic of exosomes is their endosomal origin, which distinguishes them from apoptotic bodies and microvesicles that are formed through the inward budding of the endosomal plasma membrane [31,32], leading to the formation of late endosomes and multivesicular bodies (MVBs) inside the cell. These MVBs can either fuse with the lysosome complex for degradation or they are translocated to the plasma membrane where they fuse with the cell membrane and exit the cell to release their intraluminal vesicles as exosomes into the extracellular space [33,34,35]. The Endosomal Sorting Complex Required for Transport (ESCRT) protein plays a key role in orchestrating the cargo loading and vesicle release [36]. This involves binding large multi-subunit protein complexes to ubiquitinated proteins, inducing the inward budding of the endosomal membrane. Each complex within the ESCRT pathway contains multiple ubiquitin-binding domains, which are responsible for sorting ubiquitinated proteins into intraluminal vesicles (ILVs) and determining the cargo of exosomes. On the other hand, there also exists an alternate exosome pathway that is ESCRT-independent and is regulated by phosphorylated RAB31. The alternative pathway employs flotillin proteins in lipid raft microdomains that prevent the fusion of the multivesicular endosomes with lysosomes and instead enables the secretion of the ILVs outside of the cells as exosomes [37].

The release of EVs from microglia is accelerated by various factors that often precede the activation of the parent microglia and are usually associated with environmental changes or cellular stress. Some of the key triggers include cytokines (TNF-α, IL-1β, IFN-γ), chemokines, lipopolysaccharides (LPSs), oxidative stress (ROS), oxygen deprivation or hypoxia, glutamate, serotonin, and adenosine triphosphate (ATP) [17]. A well-studied example that potentiates the release of MGEVs under normal physiological conditions is serotonin, which is released by neurons. Serotonin activates 5-HT2a, 5-HT2b, and 5-HT4 serotonin receptors on the parent microglia cells. The activation of these receptors elevates intracellular calcium levels, promoting the fusion of multivesicular bodies (MVBs) with the plasma membrane with a subsequent release of the EVs by these glial cells [17]. Another potent inducer of exosome secretion from microglial cells is ATP, which is released by damaged or stressed neurons. Elevated concentrations of extracellular ATP present within the microenvironment of microglial cells bind to the purinergic receptors on microglia, leading to their activation and subsequent MGEV release [38]. Both in vitro studies and in vivo experiments conducted by Verderio et al. (2012) in mice with subclinical neuroinflammation demonstrated that MGEVs produced following ATP stimulation triggered a much stronger inflammatory response in activated microglia [39]. Analysis of the proteome of MGEVs released during ATP-induced microglial activation revealed that ATP drives the sorting of proteins involved in cell adhesion, extracellular matrix (ECM) organization, and the autophagy–lysosomal pathway, and enzymes associated with cellular metabolism, which regulate the function of recipient astrocytes and neurons within the local microenvironment [39].

### 2.2. Cargo Composition and Regulation of Cargo Selection in MGEVs

MGEVs contain a diverse array of biologically active cargo molecules. When MGEVs are taken up by recipient cells, these cargoes modulate their function and thus play a crucial role in intercellular communication within the CNS [40]. The cargo composition of MGEVs includes a variety of the molecular constituents of their parent cell of origin, consisting of proteins, lipids, and nucleic acids, including noncoding RNAs, each contributing to diverse biological effects. In general, EVs comprise phospholipid bilayers of phosphatidylserine, phosphatidylcholine, sphingomyelin, ceramides, and cholesterol that combine to maintain the structural integrity and function of these nanovesicles [15]. The EV surface is decorated with various antigens and proteins which interact with corresponding receptors on target cells to trigger specific intracellular signaling pathways [41]. EVs also carry glycoproteins, glycolipids, and signaling molecules including cytokines, growth factors, and an array of metabolites [41].

#### 2.2.1. MGEV Cargo Reflects Parent Cell Phenotype

The cargo composition of MGEVs is largely determined by the physiological or pathological state of the parent microglial cell, and this information is preserved relative to its origin and the specific stimuli from the surrounding microenvironment [42,43,44,45]. Therefore, ultimately, the microglial phenotype, whether detrimental or beneficial, is the critical factor driving the cargo content of the secreted exosomes and thus their functional effects, including those associated with maintaining microglial quiescence, synaptic pruning, and anti-inflammatory activities [39,42,46] (Figure 1). MGEVs released from pro-inflammatory microglia exhibit cargoes crucial for driving neuroinflammation and the pathological progression of disease or injury. These MGEVs have been shown to carry significant levels of pro-inflammatory cytokines, including TNF-α and interleukin (IL)-1β, as well as miRNAs that lead to reduced spine and synaptic density in neurons, contributing to synaptic dysfunction, which was demonstrated through cell-based assays as well as validated in experimental murine models [38,47]. MGEVs derived from pro-inflammatory microglia play an important role in PD by promoting α-synuclein aggregation pathology [25]. It was shown that MGEVs derived from the BV2 microglial cell line primed with pro-inflammatory mediators, such as LPSs, exhibited TNF-α and IL-6 in their cargoes [48,49]. Conversely, studies have also demonstrated that when microglia are exposed to agents that polarize them towards a beneficial phenotype, the released MGEVs are functionally beneficial. MGEVs from IL-4-expressing BV2 cells stimulate the production of anti-inflammatory ARG1 and YM1 when taken up by tissue microglia in a murine model of multiple sclerosis (MS) to elicit neuroprotective effects [50]. These beneficial MGEVs, when internalized by neurons both in vitro and in vivo in mouse models of middle cerebral artery occlusion, support neuron survival due to the transport of pro-survival noncoding microRNAs such as miR-124 [51]. Similarly, another study showed that MGEVs generated from microglia that were modulated with 1070 nm light provided an improvement in the cognitive function of an AD mouse [52]. In other work, an omega-3 polyunsaturated fatty acid (PUFA) stimulus of microglia has been shown to alter microglial reactivity and promote the release of MGEVs containing nerve growth factor (NGF) that can activate the protective NGF/TrkA pathway to reduce apoptotic neuronal death both in and TBI model mice [53]. Despite continued research endeavors, the precise molecular mechanisms driving the selective sorting and packaging of cargoes within MGEVs have not been fully elucidated. MGEVs also selectively express complement component 1q (C1q) subunits, CD13, and the lactate transporter monocarboxylate transporter 1 (MCT-1), which serve as unique characteristic markers to specifically identify MGEVs [39,54] in addition to cargo and surface proteins like CD14, IL-1β, TMEM119, and CD11b [55].

Work has also been initiated to examine the cargo content of MGEVs derived from human microglia. Recent research comparing two different immortalized human microglial cell lines, HCM3 cells derived from human embryonic microglia and C20 cells obtained from human adult microglia, revealed a stark difference in the miRNA cargoes of their exosomes. These two cell lines represent opposite ends of the inflammatory spectrum with different basal activation states and distinct capacities to influence glioblastoma proliferation. Such differences were attributed to variations in the miRNA signature of their EVs, which are reflective of their polarization states under resting conditions. Gene expression analysis showed that C20 cells were in a more pro-inflammatory state compared to HMC3 cells, with the latter exhibiting significantly higher levels of IL-4 and lower levels of TNF-α and IL-6 gene expression [56,57]. Several factors, such as age and sex [58,59,60,61], which influence the activation state heterogeneity of microglial cells, are likely also crucial in modulating the cargo and the associated functions of MGEVs, influencing whether they contribute to neuroprotection or neurodegeneration.

#### 2.2.2. Microglial Location in the CNS Influences EV Cargo Composition

The location of microglia in the CNS appears to influence both the cargoes they contain and their biological effects. In studies by Murgochi and colleagues (2020), it was shown that MGEVs from primary microglia in culture, isolated from different CNS regions, carried out distinct biological functions [62]. Although morphologically similar, cortical microglia isolated from the cortex of neonatal rodent pups were found to be more neuroprotective and associated with neurogenic and tumorigenic properties, while conversely, MGEVs released by spinal cord microglia were more pro-inflammatory and were able to significantly mitigate glioma proliferation following LPS stimulation. Pathway enrichment analyses following the proteomic profiling of the donor microglia from the two regions indicated that cortical microglia are more closely linked to neuronal migration, neurogenesis, and monocyte recruitment under basal conditions, while spinal cord microglia are predominantly associated with Na+/H+ antiporter activity, nerve degeneration, and lipid degradation. Similarly, proteomic analyses of MGEVs released from microglial cells from both CNS locations revealed pronounced differences in their cargo composition which was reflected in their unique biological functions, suggesting a distinct functional dichotomy where EVs from spinal cord microglia were primarily linked to inflammation, while those from cortical microglia were associated with nerve regeneration [62].

#### 2.2.3. Signature Proteins Associated with MGEVs

Proteomic analysis has identified a variety of proteins within MGEVs that can participate in both physiological and pathological processes. The main proteins found in MGEVs, irrespective of the cell phenotype of origin, include those crucial for membrane transport and fusion, such as Rab GTPases and annexins; proteins involved in exosome formation and multivesicular body (MVB) processing, like the ESCRT complex, ALIX, and TSG101; heat shock proteins including HSC70, HSP60, Hsp70, and Hsp90; integrins; the tetraspanin family members CD9, CD63, CD81, and CD82; various cytoskeletal proteins including actin, tubulin, cofilin, profilin, and fibronectin; signaling proteins; immune regulatory molecules (MHC I and II); and various metabolic enzymes [63,64]. However, when microglial homeostasis is disrupted by pro-inflammatory stimuli, MGEVs undergo significant changes in their cargo, exhibiting in addition pro-inflammatory cytokines (e.g., IL-1β, TNF-α), chemokines (e.g., CCL2, CXCL10), and enzymes (e.g., MMP-9) that contribute to extracellular matrix (ECM) remodeling. In contrast, under anti-inflammatory conditions, MGEVs carry growth factors such as BDNF which promote neuroprotection, repair, and cell survival in the inflamed mouse brain [65,66]. Further work is needed to explore these effects in detail.

#### 2.2.4. Lipidome of MGEVs

Distinct lipid profiles have been identified in EVs originating from various cell sources of the CNS [67], and recent investigations indicate that shifts from normal to pathophysiological cell states can significantly alter the lipid composition of EV membranes. Exosomes released from neurons and glial cells are predominantly enriched with ceramides and glycosphingolipids, including gangliosides [67]. A comparison of four lipid types—glycerophospholipids, sphingolipids, glycerolipids, and sterol lipids—between frontal cortex tissue and brain-derived EVs showed that EVs had higher glycerophospholipid levels but lower levels of sphingolipids. Notably, there was a strong enrichment of glycerophosphoserine and lower amounts of glycerolipids and cholesterol esters. This suggests that different brain regions produce EVs with distinct lipid compositions, potentially influencing their function and membrane fluidity [68], which in turn can modulate cellular signaling pathways and immune responses in the recipient cells [69]. Lipidomic analyses of MGEVs have revealed the presence of various lipid species including cholesterol, sphingomyelin, and phosphatidylserine. Sphingolipids present in MGEVs have been shown to influence amyloid-β clearance, suggesting a role in neurodegenerative diseases [69]. In a recent study investigating the lipidomic profile of purified small MGEVs from cryopreserved postmortem human parietal cortex tissues, comparing age-matched late-stage AD to normal or low-pathology cases identified a unique lipid profile with 594 distinct lipids that primarily reflected pro-inflammatory characteristics and identified specific lipids associated with endolysosomal dysfunction [70]. The study also found a notable AD-related decrease in DHA-containing polyunsaturated lipids, along with elevated levels of free cholesterol [70]. Despite significant progress in MGEV lipidomic technologies, further research is needed to fully characterize the lipid content of EVs from various microglial phenotypes and understand their potential impact on different CNS-related pathological conditions.

#### 2.2.5. Nucleic Acid Composition of MGEVs

MGEVs also carry nucleic acids, including microRNAs (miRNAs), messenger RNAs (mRNAs), and even genomic DNA, both within the vesicle and upon its surface. These nucleic acids can be transferred to target cells, where they regulate gene expression and cellular functions. Apart from mRNAs and miRNAs, MGEVs also contain ribosomal RNAs (rRNAs), long noncoding RNAs (lncRNAs), transfer RNAs (tRNAs), small nuclear RNAs (snRNAs), small nucleolar RNAs (snoRNAs), and p-element-induced wimpy testes (piwi)-interacting RNAs (piRNAs) [40,71]. These EV RNAs are currently gaining interest as powerful biomarkers due to their stability, recipient cell specificity, and ability to reflect the molecular state of the parent cells, offering a noninvasive and accurate tool for the early diagnosis and monitoring of neurological diseases [15,72]. In addition to the different RNA species, EVs also carry double-stranded (ds) or single-stranded (ss) DNA, as well as mitochondrial DNA (mtDNA) [73,74].

#### 2.2.6. mRNA Content in MGEVs

Recent studies by Ransom et al. (2024) investigated mRNAs in small extracellular vesicles (sEVs) from both healthy and Alzheimer’s disease-affected human brains as well as from murine primary cultures of different cellular origins, and identified more than 10,000 intact, full-length polyadenylated mRNA transcripts in these nanovesicles, with the potential to generate functional proteins upon translation. The mRNAs within these sEVs showed considerable isoform diversity and were selectively packaged based on the parent cell type and disease state [75]. It was found that MGEVs were enriched with mRNAs associated with cytokine production, immune response, and adaptive immunity, including increased interferon gamma and beta response transcripts, along with various chemokine ligands and receptors. Similarly, *ApoE* transcripts were detected in sEVs from different neural sources within the Alzheimer’s brain, with notably higher levels in microglial sEVs. Additional prominent transcripts identified in MGEVs included MS4A4A and MS4A6A, both linked to neuroinflammatory responses [75]. These findings suggest that the transcriptome of human brain sEVs is enriched with diverse mRNA cargoes that are significantly altered in Alzheimer’s disease. This enrichment and dysregulation in mRNA transport via MGEVs may play a pivotal role in the propagation of AD and other neurodegenerative conditions by affecting key cellular processes.

#### 2.2.7. miRNA Profiles Shape MGEV Functions in the CNS

MicroRNA profiles within MGEVs are crucial in defining their function within the CNS. Specific miRNAs modulate pathways involved in neuroinflammation, cell survival, and synaptic plasticity, allowing MGEVs to influence cellular responses in nearby neurons and glial cells within their microenvironment. This regulatory capability positions MGEV miRNAs as key players in both protective and pathogenic roles in the context of neurotrauma and degenerative diseases [76].

*Pathogenic Impact of MGEV miRNAs:* MicroRNAs in MGEVs can play a crucial pathogenic role in the CNS by influencing neuroinflammatory and neurodegenerative responses. These miRNAs target genes involved in the immune response, synaptic function, and cellular stress pathways, potentially leading to increased neurotoxicity, reduced synaptic plasticity, and dysregulated immune responses in recipient cells [17]. By transferring these miRNAs, MGEVs contribute to the spread of neuroinflammation, amplifying pathology across neural networks and potentially accelerating disease progression [47,77,78]. For instance, the pathogenic MGEV miRNA miR-155 is known to modulate inflammatory signaling pathways, potentially exacerbating the pathophysiology of neurodegenerative conditions like Alzheimer’s disease and multiple sclerosis [79,80].

Pro-inflammatory MGEVs have also been found to confer a proangiogenic ability in resting microglia [56] via miR-155-5p that is present in their exosome cargo. MGEV-derived miR-155-5p has been demonstrated to suppress SOCS1, resulting in an upregulation of the NFκB pathway, which consequently triggers the pro-inflammatory signaling cascade as well as driving the proangiogenic effect that occurs through the Irf1/miR-155-5p/Socs1 signaling pathway [43]. These pro-inflammatory MGEVs therefore promote the activation of resting microglia as well as amplifying proangiogenic effects contributing to the pathophysiology of neovascular retinal diseases [56].

Several studies have correlated a dysregulated expression of miR-146a, which is abundantly expressed in the CNS, with multiple neurodegenerative diseases, thereby making it a potential biomarker and a crucial treatment target [80,81]. Recent investigations by Fan et al. (2022) indicated that dysregulated levels of MGEV-enriched miR-146a-5p inhibited neurogenesis and the spontaneous discharge of excitatory neurons by targeting Krüppel-like factor 4 (KLF4) in a rodent model of depression [55]. Reducing miR-146a-5p expression in these experiments mitigated adult neurogenesis deficits in the dentate gyrus (DG) associated with this neuropathological condition. Evidence of the deleterious effects of miR-146a was also shown by Zhou et al. (2016), who found that the downregulation of miR-146a led to neuroprotective effects, which occurred via targeting pro-apoptotic genes like Caspase7 and Bclaf1 in in vitro models of cerebral ischemic injury [82]. Similarly, inflammatory MGEVs carrying elevated levels of miR-146a-5p were demonstrated to downregulate synaptotagmin1 (Syt1) and neuroligin1 (Nlg1) expression in recipient neurons that contribute to important functions in dendritic spine formation and synaptic stability, which when downregulated resulted in the decreased density and strength of excitatory synapses [47]. 146a-5p was also one of the critical miRNAs that was identified to be significantly upregulated in epilepsy patients compared to healthy controls [83]. Apparently, both miR-155 and miR-146a are upregulated in the lumbar spinal cord of rodent EAE models of MS, which are recognized as key biomarkers of human MS [84]. Another example of the pathogenic effects of miRNAs packaged within MGEVs is miR-466i-5p, which has been shown to induce brain injury by promoting apoptosis in hippocampal neurons in mouse models of heatstroke [85].

*Protective Role of MGEV miRNAs:* MGEV miRNAs play a protective role within the CNS by modulating anti-inflammatory and neuroprotective pathways. Specific miRNAs like miR-124 have been shown to reduce neuroinflammation, promote neuronal survival, and enhance synaptic function [86,87]. It was demonstrated that MGEVs containing elevated levels of miR-124-3p conferred neuroprotective effects in mouse models of repetitive TBI by targeting focal adhesion kinase family interacting protein of 200 kDa (FIP200)-mediated neuronal autophagy. This intervention led to reduced modified neurological severity scores (mNSS) and significant improvements in performance on the Morris water maze (MWM) test, indicating miR-124-3p is able to mediate an improvement in cognitive recovery after TBI [88]. Similarly, other studies have demonstrated, using in vivo models of CNS injury, that miRNAs within MGEV cargo such as miR-124-3p could reduce neurodegeneration and improve cognitive function after TBI [86]. This reduction in neuroinflammatory responses occurred due to the inhibition of mTOR signaling by the miR-124-3p-mediated downregulation of PDE4B and the targeting of the RelA/ApoE signaling pathway [86,89]. In addition, miR-124-3p-containing MGEVs also supported neurorepair and plasticity. RNA sequencing and functional assays in vitro and in vivo confirmed that miR-124-3p-loaded MGEVs transformed microglia into a beneficial phenotype, suppressing mTOR signaling and enhancing autophagy in brain microvascular endothelial cells (BMVECs), which in turn led to improved BBB integrity and neurological outcomes in experimental TBI [90]. Interestingly, aside from inhibiting neuroinflammation, MGEVs containing miR-124-3p have also been shown to promote neurite outgrowth in vitro in a scratch injury assay and improve neurological outcomes in mice after repetitive mild TBI [86,89]. MGEVs carrying miR-124-3p, derived from alternately activated, pro-reparative microglia, were also found to regulate ferroptosis in HT22 cells under oxygen–glucose deprivation and reoxygenation (OGD/R) in cell-based assays [91]. Conversely, MGEVs deficient in miR-124-3p lacked these neuroprotective effects. Further investigations also revealed that miR-124-3p-loaded MGEVs target and regulate nuclear receptor coactivator 4 (NCOA4) to prevent OGD/R-mediated cytotoxicity [91].

miR-124-carrying MGEVs therefore hold significant therapeutic potential and appear to be enriched following the IL-4 priming of the parent microglia. Recent studies employing murine models of ischemic stroke have confirmed that miR-124 negatively affects astrocytes in the context of post-stroke recovery [92]. The systemic administration of these IL4-polarized MGEVs suppressed astrocyte proliferation and migration, which are important cell behaviors associated with the formation of glial scars. By promoting the dedifferentiation of astrocytes, these modified MGEVs significantly reduced astrogliosis. Mechanistically, this effect occurred at the level of transcription, involving the suppression of the transcription factor Signal Transducer and Activator of Transcription 3 (STAT3), which contributes to the induction of astrogliosis, by MGEV-derived miR-124 [93]. The anti-scarring role of these MGEVs was further validated by miR-124 knockdown, which inhibited the beneficial effects [92]. Other studies have demonstrated that MGEVs released from IL-4-polarized anti-inflammatory microglia carrying elevated levels of MiR-124 downregulate the neuroinflammatory cascade by targeting the TLR/NFκB pathway [94].

Other miRNAs found in MGEVs that have shown beneficial effects in models of neurotrauma or neurodegenerative disorders include miRNA-137, which has been demonstrated to reduce ischemic brain injury in murine models of transient middle cerebral artery occlusion (tMCAO) by targeting Notch1 signaling pathways [95]. miR-135a-5p, on the other hand, has been found to suppress neuronal autophagy in a mouse model of transient middle cerebral artery occlusion (tMCAO) by negatively regulating the expression of thioredoxin-interacting protein (TXNIP) and preventing the activation of the NLRP3 inflammasome to confer a neuroprotective effect [96]. In this context, the systemic administration of the MGEVs for 2 days after MCAO significantly reduced BBB disruption in a rodent model of cerebral ischemia [97]. The therapeutic effect was found to be mediated by miR-23a-5p, which downregulated NFκB p65 expression and led to ensuing decreases in TNF-α and MMP3. Changes in neuroinflammatory mediators correlated with a marked reduction in brain infarct and edema volume, an improvement in neurological scores, and an increase in the expression of ZO-1, occludin, and claudin-5 after MCAO. Knocking down miR-23a-5p, on the other hand, was found to reverse these beneficial effects [75]. Lastly, studies by Tian et al. showed that MGEVs from IL-4-treated microglia could accelerate angiogenesis both in in vitro assays as well as following acute MCAO. Proangiogenic effects were attributed to the presence of significantly elevated levels of miRNA-26a in these MGEVs as compared to MGEVs from LPS-polarized or -unpolarized microglia [56].

Interestingly, MGEVs derived from anti-inflammatory microglia have been shown to contain miR-146a-5p, an miRNA that is associated with mitigating cognitive deficits induced by intermittent hypoxia, a key predictor of cognitive decline in AD patients with obstructive sleep apnea. MGEVs carrying miR-146a-5p are also able to regulate the NLRP3 inflammasome and reduce neuroinflammation in mice exposed to intermittent hypoxia [98]. It was found that miR-146a-5p modulates mitochondrial reactive oxygen species (ROS) by targeting HIF1α, impacting the NLRP3 inflammasome and associated neuroinflammation. miR-146a has been shown to contribute to modulating the neuroinflammatory response in multiple neurological diseases by regulating the common inflammation-related target genes such as IRAK1, TRAF6, and CFH and associated pathways such as TLR signaling and complement activation, suggesting that miR-146a-5p is a potent inhibitor of neuroinflammation [81]. The role of MGEVs carrying miR-146a-5p in the CNS therefore appears to be complex and controversial, displaying a differential effect that can be either protective or pathogenic. This miRNA can play a role in neuroinflammation, potentially aggravating disease conditions, yet it also has regulatory effects in several neurodegenerative diseases, supporting anti-inflammatory responses and neural repair and regeneration, producing myelin protein, and exerting anti-apoptotic and neuroprotective effects [81]. This dual functionality of miR-146a underscores its complex role in the CNS.

A number of recent investigations have highlighted the role that the donor source for EV derivation plays in their therapeutic effects on the rate of recovery after spinal cord injury (SCI) [99]; however, similar work with MGEVs remains less explored [100,101] when compared to mesenchymal stem cells (MSCs) and Schwann cells (SCs) [102,103,104]. A bioinformatics analysis of primary MGEVs that exhibited neuroprotective effects in an acute mouse model of spinal cord contusion revealed elevated levels of miRNA-151-3p associated with their cargo, which has P53 as one of its specific targets. The MGEV-mediated transfer of miR-151-3p was found to play a key role in inhibiting neuronal apoptosis and promoting axonal growth through the inhibition of the p53/p21/CDK1 signaling pathway. Therefore, miRNA-151-3p appears to be a key component of MGEVs, contributing to their neuroprotective effects following SCI [105,106]. These protective actions make MGEV miRNAs valuable in maintaining neural health and counteracting the effects of neurodegenerative diseases.

### 2.3. Mechanisms of Exosome Uptake

MGEVs can interact with a variety of cell types within the CNS, including neurons, astrocytes, oligodendrocytes, and other microglia. Through these interactions, they play an important role in modulating the CNS environment [107]. The interaction and uptake of exosomes in recipient cells occurs through various mechanisms [108], including fusion, phagocytosis, pinocytosis, and direct surface contact. MGEV uptake by target cells primarily includes clathrin-mediated endocytosis, micropinocytosis, uptake via lipid raft-associated endocytosis, and direct fusion with the plasma membrane. Clathrin-mediated endocytosis and micropinocytosis are the primary means of EV uptake in microglia. In the former method, exosomes adhere to the plasma membrane and are subsequently internalized into endosomes where they are either degraded in the lysosome or may escape the lysosomes to release their cargoes into the cytoplasm. Macropinocytosis, on the other hand, which involves the plasma membrane forming ruffles in an actin-dependent process to internalize extracellular substances, is also a major means of exosome uptake in microglia, a process that is normally constitutive in these glial cells [109]. Exosome uptake is a more selective process that is driven by the recipient cell type. Recent research indicates that, under healthy physiological conditions, astrocytes are unable to internalize exosomes from oligodendrocytes or microglia [110]. Microglia, on the other hand, have been reported to effectively internalize EVs released from a wide range of neural cell types [107,109,111].

### 2.4. Role of Exosomes in Intercellular Communication and the Mechanism of Action of MGEVs

The mechanisms by which MGEVs exert their effects is similar to EVs from other cell types and involves the direct transfer of bioactive molecules to recipient cells, the modulation of receptor-mediated signaling pathways, and gene expression changes driven by exosomal miRNAs and other regulatory RNAs [7,112]. MGEVs transfer their cargo constituting miRNAs or proteins that regulate inflammatory signaling cascades in the recipient cells, either by amplifying or dampening neuroinflammatory responses [17,86,90,113,114]. In neuropathological conditions, the role of MGEVs in intercellular communication is important. MGEVs can convey signals that either promote the clearance of insoluble protein aggregates, such as amyloid-beta (Aβ) in Alzheimer’s disease [115], or propagate the spread of pathogenic proteins, such as tau or α-synuclein [25,116,117]. MGEVs, like exosomes from most other cell types, can cross the blood–brain barrier (BBB) [118,119,120]. Using in vitro models, it was demonstrated that MGEVs from reactive microglia dramatically enhanced the permeability of the BBB while reducing the expression of BBB-related proteins like Claudin-1, occludin, and ZO-1. In contrast, MGEVs from resting microglia did not alter the integrity of the BBB [120]. Thus, MGEVs not only mediate local immune responses but, due to their ability to cross the BBB, can affect target cells at a distance and systemically or even be employed as vehicles for delivering therapeutic agents to the brain.

## 3. Beneficial Effects of Microglial Exosomes in Neurological Injury and Disorders

Recent studies show that MGEVs are versatile mediators of intercellular communication within the CNS and can be employed to impart beneficial effects on target cells and tissues [112]. MGEVs contribute to neural homeostasis by enhancing synaptic activity through promoting sphingolipid metabolism [18] and regulate the extracellular matrix (ECM) via the delivery of surface-decorated enzymes to promote tissue remodeling and repair. Thus, MGEVs can either maintain ECM integrity or facilitate its degradation, depending upon their cargoes [121]. Work has also shown that MGEVs are crucial for microglia–microglia communication, autophagy activation, and the modulation of inflammatory responses [122], as well as being involved in the processes of demyelination and remyelination [66]. The proteomic analysis of MGEVs has provided concrete evidence that they can carry neuroprotective cargoes capable of modulating neuroinflammation, promoting synaptic plasticity, facilitating the clearance of deleterious protein aggregates, and protecting neurons from oxidative stress, thus making MGEVs important agents for maintaining CNS homeostasis under conditions of stress or injury [22,123]. Understanding the mechanisms that govern this dichotomy in MGEV function in recipient cells will offer valuable insights into novel MGEV-based therapeutic strategies for neurodegenerative diseases [122]. In this section, we will elaborate on the underlying mechanisms by which MGEVs contribute to beneficial effects after CNS injury and disease.

### 3.1. Modulation of Neuroinflammation

Contrary to the pro-inflammatory role of MGEVs under specific pathological conditions, MGEVs can also exert neuroprotective effects through their delivery of anti-inflammatory cytokines (e.g., IL-4, IL-10) and immunomodulatory molecules that suppress excessive immune responses in the CNS [22,124,125]. This immunomodulatory function of MGEVs helps to mitigate neuroinflammation and attenuate neuronal damage as well as promote neuronal survival and repair [66] in various neurological disorders. The ability of MGEVs to either attenuate or amplify inflammatory responses in the CNS depends on their cargo. In the context of neuroprotection, MGEVs carrying miRNAs, such as miR-146a and miR-124 [55,86,126], have been shown to reduce the expression of pro-inflammatory cytokines, with the MGEVs acting through the negative regulation of the TRAF6/NF-κB signaling axis [127,128,129] and inhibiting the activation of the NLRP3 inflammasome [98]. A study by Ge et al. (2020) [126] demonstrated that MGEVs enriched with miR-124-3p alleviated neurodegeneration and improved cognitive function in TBI [86]. These miRNA species were shown to target TLR4/MyD88/NF-κB p65 signaling, modulate the inflammatory response, and reduce TBI-associated damage.

In addition to protective miRNAs, MGEVs can also deliver neurotrophic factors and antioxidants to neurons. MGEVs from activated microglia can contain brain-derived neurotrophic factor (BDNF), nerve growth factor (NGF), and glial cell-derived neurotrophic factor (GDNF), which are known to promote neuronal survival, enhance synaptic plasticity, and support axonal regeneration [130,131]. A recent study found that following MCAO, TGFβ carried by intravenously administered MGEVs promoted neuronal survival, stimulated angiogenesis, and shifted activated microglia towards a beneficial phenotype in ischemic brain tissue by activating the SMAD-2/3 pathway [132]. MGEVs also carry proteins and enzymes within their exosome cargo that help to reduce oxidative stress in the CNS. Peng et al. [100] conducted studies using a mouse model of SCI, demonstrating that MGEVs helped to mitigate oxidative stress and prevent endothelial dysfunction, while promoting the restoration of endogenous microvascular repair following SCI. It was subsequently demonstrated that these MGEVs promoted the survival, regeneration, and function of endothelial cells by modulating the Keap1/Nrf2/HO-1 signaling axis, which resulted in an increase in downstream antioxidative-related genes including *NQO1*, *Gclc*, *Cat*, and *Gsx1* as well as levels of the transcription factor Nrf2 and heme oxygenase 1 (HO-1), which are critical regulators of oxidative stress in cells [133]. Similarly, modified MGEVs derived from IL-4-primed parent microglia reduced pericyte apoptosis, promoted vascular remodeling, and enhanced endothelial cell proliferation, resulting in decreased vascular leakage in rodent models of diabetic retinopathy [134]. These MGEVs not only decreased neuronal impairment and mitochondrial dysfunction in experimental AD models [135] but also provided neuroprotection by reducing A1 astrocyte activation, leading to motor function recovery in rodent models of spinal cord injury (SCI) [101].

### 3.2. Promotion of Neurogenesis, Synaptic Plasticity, and Repair

**Figure 3:**
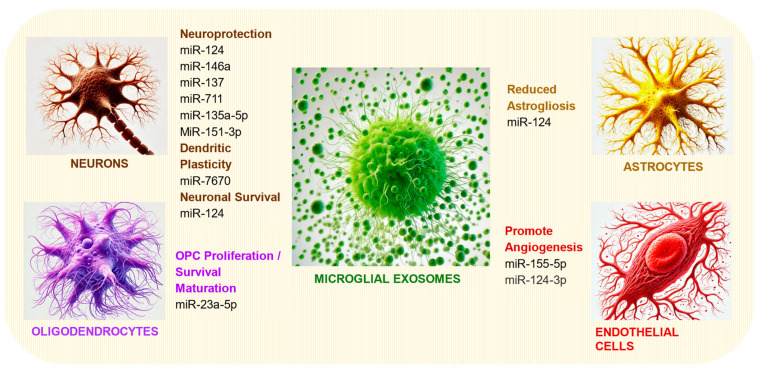
miRNA-driven beneficial roles of microglial exosomes in the CNS.

In addition to their support of neuronal survival, MGEVs have also been demonstrated to promote neurogenesis and synaptic plasticity through their miRNA cargoes [18,47,77] (Figure 3). Previous studies by Antonucci et al. (2012) [18] demonstrated that microglia-derived microvesicles play a key role in microglia-to-neuron communication by releasing glutamate, which modulates synaptic activity at presynaptic sites. In animal studies, injecting MGEVs into the visual cortex of rats led to an acute increase in excitatory transmission following visual stimuli that was attributed to enhanced sphingolipid metabolism [18].

A few studies have also demonstrated that synaptic plasticity, essential for learning, memory, and cognitive function, is often impaired in neurodegenerative diseases such as AD and PD [136,137]. MGEVs carry not only miRNAs but also synaptic proteins that are vital for maintaining synaptic integrity and function, such as synapsin, a protein associated with synaptic vesicles involved in neural development, and these MGEVs have been found to be released primarily during periods of either increased neuronal activity or cellular stress [138]. The MGEV-induced production of ceramide and sphingosine can elevate neuronal sphingolipid metabolism, driving increased synaptic activity in recipient neurons and increased excitatory transmission [18,138,139]. This capacity to enhance synaptic plasticity and repair makes MGEVs a promising therapeutic approach for restoring cognitive function in neurodegenerative diseases. These findings underline the significance of MGEVs in regulating synaptic activity and their critical role in microglia-to-neuron signaling pathways [18].

MGEVs from differentially activated microglia can also affect inhibitory synaptic transmission. Prada et al. (2018) showed that MGEVs released from inflammatory microglia led to the loss of excitatory synapses. This effect is associated with the transfer of miR-146a-5p, a microglia-specific miRNA that regulates presynaptic synaptotagmin1 (Syt1) and postsynaptic neuroligin1 (Nlg1), both of which are essential for dendritic spine formation and synaptic stability [47]. Other works have suggested that endocannabinoids, known to influence neuronal synaptic communication in the CNS, are transported via MGEVs. These vesicles carry N-arachidonoylethanolamine (AEA) on their surface, which activates type 1 cannabinoid receptors (CB1s), leading to the inhibition of presynaptic transmission in GABAergic neurons [140]. Further research is needed to determine whether the MGEV modulation of neurotransmission is critical for maintaining normal homeostasis or if it contributes to neuropathological conditions by disrupting the balance between excitatory and inhibitory transmission.

### 3.3. Clearance of Deleterious Protein Aggregates

MGEVs play a beneficial role in clearing neurotoxic proteins such as amyloid-β in AD and α-synuclein in PD [69]. While more research is needed to confirm the direct involvement of MGEVs in the phagocytic clearance of harmful protein aggregates, evidence suggests that MGEVs contribute to not only the propagation of Aβ aggregation and neuroinflammation, but also aid in the removal of neurotoxic proteins [141]. In AD, MGEVs have been shown to clear amyloid-β peptides by delivering insulin-degrading enzyme (IDE), which prevents Aβ aggregation and plaque formation [142]. The clearance is regulated by proteins such as Glutaminase C (GAC) and TREM2, which promote exosome release and facilitate the microglial phagocytosis of amyloid-β [143,144]. A protective role for TREM2 in reducing exosomal tau pathogenicity has been proposed, with studies showing that the dysfunction or loss of TREM2 expression leads to an increased spread of tau pathology driven by MGEVs [116]. These findings emphasize the beneficial impact of MGEVs in mitigating neurodegenerative diseases by potentiating the clearance of toxic protein aggregates.

### 3.4. Regeneration and White Matter Repair

The regenerative capacity of MGEVs has been demonstrated across species from leeches to rodents, where they act as a vehicle for transferring pro-regenerative molecules [50,145]. Evaluation of the cargo content of MGEVs from these different sources has identified neurotrophic factors, including TGF-beta and prosaposin [123,146,147,148], that are capable of inducing neurite outgrowth in dorsal root ganglion (DRG) neurons [62], leech neurons [146], and rat embryonic cortical neurons [123], as well as promoting differentiation in PC12 cells [146]. MGEVs also contain cargoes that modulate key metabolic processes involved in axon growth and guidance, dendrite development, and filopodium assembly. In studies with leech microglia, the neurotrophic effects of MGEVs were partially mediated by nGDF (nervous Growth/Differentiation Factor), which is a member of the TGF-β family [123,146,147,148]. Additionally, the cargo evaluation of MGEVs from the N9 microglial cell line revealed the presence of metabolic enzymes that are associated with anaerobic glycolysis and lactate production [39,54], suggesting that MGEVs may serve as reservoirs of energy substrates that are essential for neurite outgrowth.

MGEVs from pro-regenerative microglia actively support myelin repair, and conversely MGEVs generated from inflammatory microglia have been shown to hinder remyelination. Remyelination failure in the CNS is associated with many neurological disorders, such as multiple sclerosis. Lombardi et al. [145] conducted a comparative study to evaluate the effects of MGEVs from either pro-inflammatory or pro-regenerative microglia on oligodendrocyte progenitor cell (OPC) function in a mouse model of lysolecithin-induced demyelination in the corpus callosum. It was found that MGEVs from microglia with regenerative phenotypes significantly enhanced OPC recruitment, migration, differentiation, and myelin repair, whereas MGEVs from pro-inflammatory microglia hindered remyelination. The beneficial effects of these MGEVs were attributed to their surface lipid component, sphingosine 1-phosphate, which played a key role in promoting myelin repair [145]. MGEVs have also been demonstrated to promote the migration and differentiation of OPCs into oligodendrocytes and contribute to myelin regeneration as well as aid in white matter repair to reverse cognitive impairment [92]. The presence of miR-23a-5p in MGEVs has been identified as a key factor in driving OPC migration, differentiation, and myelination [66]. In other studies, miR-23a-5p in MGEVs from anti-inflammatory microglia was found to promote functional recovery in a mouse model of cerebral ischemia [149]. These findings underscore the crucial functional role of MGEVs in OPC biology and the process of myelination.

### 3.5. Reducing Oxidative Stress in Neurons

Peng et al. (2021) showed that MGEVs can improve function after SCI by inhibiting oxidative stress and promoting normal endothelial cell function. MGEVs were found to attenuate the levels of ROS and the protein NADPH oxidase (NOX2), which produces superoxide (O2•−) radicles. The positive effect of MGEVs post-SCI was demonstrated to occur through the activation of Nrf2/HO-1 signaling via the inhibition of keap1 [100]. Similarly, MGEVs derived from IL4-stimulated microglia have been shown to decrease neuronal apoptosis by reducing oxidative stress. This effect was mediated through the transfer of miR-124-3p, which modulates the ROCK/PTEN/AKT/mTOR signaling pathway to inhibit cell death [150]. Table 1 provides an overview of a number of benefecial properties associated with microglial exosomes highlighted in recent studies.

## 4. Deleterious Effects of Microglial Exosomes

**Figure 4:**
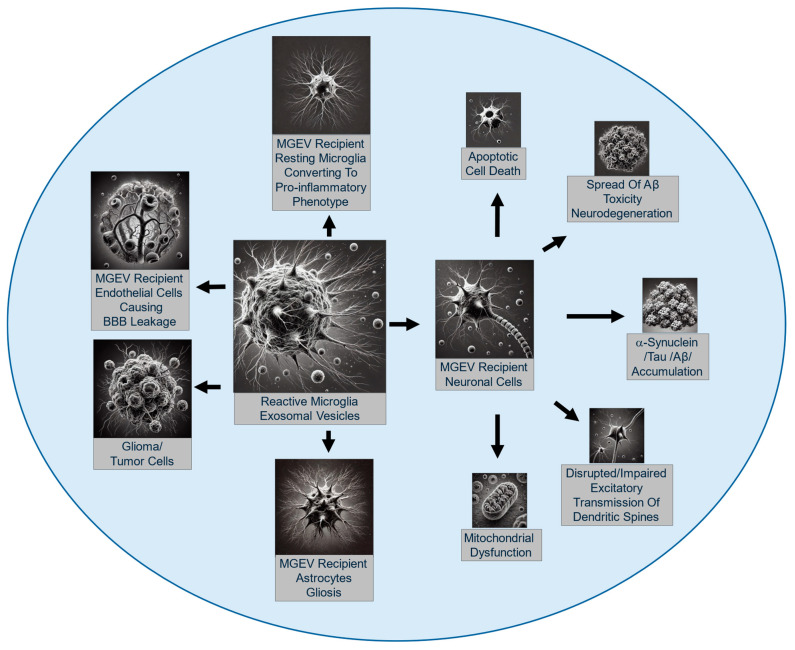
Detrimental effects of EVs released from pro-inflammatory microglia.

Although MGEVs possess the potential to protect and repair the CNS, they have conversely been associated with several neurodegenerative conditions (Figure 4). These detrimental effects are dependent upon the cargoes they carry, which can include pro-inflammatory cytokines, neurotoxic proteins, and inhibitory RNAs that exacerbate neuroinflammation, promote the spread of deleterious proteins, and impair neuronal function [16,17,154].

### 4.1. Induction of Neuroinflammation

Under pathological conditions, cytokines and chemokines within pro-inflammatory MGEVs such as IL-1β, TNF-α, IL-6, CCL2, and CXCL10 [155,156] activate recipient astrocytes within their microenvironment and drive them towards a harmful phenotype or recruit peripheral immune cells into the CNS to perpetuate neuroinflammation and exacerbate neuronal damage [16,17,46,145,155]. These MGEVs are released from microglia that are activated in response to an injury or disease and as they can also act upon the microglia that secrete them, the MGEVs can therefore create a vicious feedback loop of inflammation [17,66,157,158]. TNF-α, when present in MGEVs, can serve either a physiological function in neuronal circuit formation and synaptic plasticity to maintain homeostasis, or act pathologically to induce neuroinflammation and ensuing tissue degeneration, cell death, and myelin damage [157,159]. In recent work, it was shown that MGEVs derived from LPS-primed microglia were able to activate resting microglia in a similar fashion to direct LPS, triggering a pro-inflammatory response [40]. Similarly, MGEVs derived from TBI mice (or LPS-treated mice) have been reported to promote microglial reactivity when injected into the brains of naïve mice [160]. In vitro experiments have demonstrated that priming microglia with LPS results in the release of MGEVs loaded with elevated levels of IL-1β and miR-155, which are important pro-inflammatory mediators involved in the progression of neuroinflammation in CNS pathologies [160]. These pro-inflammatory MGEVs have been shown to contribute to numerous neurological conditions such as the worsening of stroke outcomes in aging populations [161], release amyloid-β and tau proteins into the extracellular space, and activate resting microglia and astrocytes to accelerate the exacerbation of inflammation in AD [63]. MGEVs have also been implicated in neuropathic pain following spinal nerve ligation where they trigger the IL-1β-mediated P2X7R-p38 pathway to aggravate chronic pain [162]. In HIV-associated neurological disorders, MGEVs activate the NLRP3 inflammasome, causing synaptodendritic injury and functional impairment in recipient neurons to worsen the disease [159].

In AD and PD, MGEVs are key players in the spread of misfolded proteins, amyloid-β and α-synuclein, among neurons [69,163,164], causing protein aggregation and exacerbating neuronal dysfunction to advance disease progression [69]. In neurological disease models, MGEVs have also been shown to deliver reactive radicals and other neurotoxic molecules to nearby cells within the microenvironment, leading to oxidative stress and neuronal injury [165], exacerbating cell death and synaptic dysfunction. MGEVs can influence endothelial cell function, compromising the integrity of the BBB and causing its permeability to peripheral immune cells [155] as well as other deleterious molecules from the systemic circulation. An investigation in a relapsing–remitting EAE mouse model employed the MS drug FTY720, demonstrating that it reduced EVs in the cerebrospinal fluid (CSF) after disease induction [155].

In addition to pro-inflammatory cytokines and pathogenic misfolded proteins, miRNAs in MGEVs have been recognized for their contribution to neurological disease. Pathogenic MGEVs carry miR-146a-5p, which attenuates excitatory synapses by inhibiting presynaptic synaptotagmin-1 and postsynaptic neuroligin-1, which are essential for dendritic spine stability [47]. Similarly, MGEVS that carry the endocannabinoid N-arachidonoylethanolamine on their surface can influence presynaptic transmission by modulating the endocannabinoid system by activating type 1 cannabinoid receptors on GABAergic neurons, which leads to an inhibition of presynaptic transmission [140].

### 4.2. Transmission of Pathological Proteins and Propagation of Neurodegenerative Processes

Growing evidence suggests that MGEVs transport misfolded, neurotoxic proteins and contribute to disease progression. In AD, MGEVs has been shown to facilitate the spread of Aβ peptides, leading to plaque formation and neurotoxicity, accelerating protein aggregation and neuronal dysfunction [17,69,166]. Asai et al. (2015) demonstrated that blocking MGEV release reduced tau pathology in a mouse model [28,167]. Additionally, MGEVs from AD brain tissues carry presenilin, APP fragments, and Aβ-producing proteases, promoting the formation of neurotoxic Aβ aggregates [168]. Similarly, in PD, MGEVs carry α-synuclein, the main component of Lewy bodies, spreading it to healthy neurons and promoting its aggregation, driving disease progression [25,163,164]. These studies emphasize MGEVs’ critical role in disseminating misfolded protein aggregates to nearby recipient cells via the exosome–synaptic pathway, propagating synaptic dysfunction in the CNS [25,169,170]. While MGEVs are vital for cellular communication, their ability to propagate toxic proteins poses a significant risk in advancing neurodegenerative diseases. The contribution of MGEVs to the spread of misfolded copper–zinc superoxide dismutase one (mSOD1) between cells, on the other hand, has been questioned in amyotrophic lateral sclerosis (ALS) [171,172], but direct evidence linking MGEVs to the progression of ALS has not been firmly established. A recent study has provided evidence regarding the potential correlation between pro-inflammatory microglia and dysregulated miRNAs in ALS. It has been found that activated microglia from SOD1G93A mice release certain dysregulated miRNA species such as miR-22-3p, miR-125b-5p, miR-146b-5p, miR-155-5p, miR-214-3p, and miR-365-3p that induce microglial activation and inflammatory genes in ALS [172] and can be taken up by neuronal cells in ALS [26]. Collectively, these studies provide evidence for the role of MGEVs in the transmission and propagation of several neurodegenerative conditions.

### 4.3. Contribution to Neuronal Death

MGEVs can directly contribute to neuronal death by delivering neurotoxic substances that trigger apoptosis or necrosis in recipient neurons. MGEVs carrying TNF-α and IL-6 have been shown to induce apoptosis in cortical neurons, playing a significant role in neuronal loss in neurodegeneration models [17]. Furthermore, MGEVs carrying elevated levels of reactive species can cause oxidative stress and mitochondrial dysfunction in neurons, ultimately leading to cell death. In glaucoma, where microglial cells are activated by ocular hypertension, they release EVs containing pro-inflammatory cytokines and reactive oxygen species, which induce retinal cell death [173]. In other studies, exposure to α-synuclein has been shown to activate microglial cells, leading to an increased release of MGEVs containing elevated levels of major histocompatibility complex class II and TNF-α, which contribute to neuronal death [174]. MGEVs from microglia exposed to ethanol have been shown to increase the transmission of apoptotic factors, including complement protein C1q and reactive oxygen species (ROS). These MGEVs can alter the cell membranes of β-endorphin neurons during the postnatal developmental period to induce cell death [175]. Recent research by Arvanitaki et al. (2024) shows that microglia from aged murine brains exhibit a DNA repair defect in XPF-ERCC1, leading to the accumulation of dsDNA fragments in the microglial cytosol. These fragments are packaged into MGEVs, which, when released, promote neuronal death [61]. These studies highlight how the cargo of MGEVs can have direct, deleterious effects on neuron survival, particularly when the MGEVs are derived from microglia in a pro-inflammatory or disease-activated state.

### 4.4. Impairment of Synaptic Function

Microglia play a key role in the regulation of synaptic pruning and plasticity under both normal physiological conditions like learning and memory as well as during various neurological pathologies associated with cognitive deficits linked to aging, TBI, AD, and HIV-associated neurocognitive disorder [176]. Recent studies suggest that MGEVs influence synaptic transmission, either to cause inhibition or excitation according to their cargo. MGEVs carrying toxic proteins or pro-inflammatory cytokines can disrupt synaptic integrity and signaling, leading to synaptic loss and dysfunction that contributes to cognitive decline and other neurological deficits observed in neurodegenerative diseases. In AD, MGEVs containing Aβ and inflammatory cytokines have been shown to disrupt synaptic plasticity, leading to impaired learning and memory [169]. Prada et al. (2018) suggest that inflammatory microglia release MGEVs enriched with specific miRNAs, such as miR-146a-5p. These miRNA are transferred to neurons to suppresses key synaptic proteins like presynaptic synaptotagmin1 (Syt1) and postsynaptic neuroligin1 (Nlg1), which leads to dendritic spine loss and reduced excitatory synapse density and strength, implicating MGEVs in synaptic gene silencing [47]. Support for the role of miR-146a comes from various studies that demonstrate alterations in miR-146a expression levels during the development and progression of various neurological diseases that are associated with impaired synaptic functions [47,81,177].

Recent studies by Gabrielli et al. (2022) have reported that large extracellular vesicles released by activated microglia play a pivotal role in initiating synaptic dysfunction in AD. This early synaptic impairment is a critical process that gradually extends to larger brain regions as the disease progresses. The study showed that MGEVs can alter dendritic spine density and disrupt synaptic plasticity both in vitro and in vivo, suggesting that MGEVs may drive the spread of early synaptic dysfunction in AD [169]. Work by Falcicchia et al. (2023) showed that MGEVs contribute to cortico-hippocampal network dysfunction in AD. Chronic EEG recordings revealed that a single injection of β-amyloid-laden MGEVs into the mouse entorhinal cortex was able to induce changes in cortical and hippocampal activity similar to those observed in a mouse model of AD and human AD patients. These EEG abnormalities showed a correlation with a progressive decline in cognitive function, further emphasizing the potential role of MGEVs in the propagation of AD [23]. In conclusion, MGEVs play a crucial role in both the maintenance of synaptic plasticity under normal conditions and the disruption of synaptic function in various neurological disorders. MGEVs, depending on their cargo, can either support or impair synaptic integrity. Further investigations into the role of MGEVs in synaptic regulation would provide new insights into therapeutic strategies for preventing and treating neurological diseases.

### 4.5. Inhibition of Remyelination Repair

Pathogenic MGEVs can have detrimental effects, particularly in the context of remyelination repair in demyelinating pathologies of the CNS. They have been shown to impair the function, differentiation, and maturation of oligodendrocyte precursor cells (OPCs), which are crucial for generating new myelin sheaths around demyelinated axons. MGEVs can hinder remyelination and exacerbate demyelination in conditions such as multiple sclerosis and other neurodegenerative conditions as well as contribute to the persistence of a chronic inflammatory state, further impeding the repair of myelin and worsening disease progression. Recent work has shown that MGEVs from reactive microglia have elevated levels of miR-615-5p [178], an inhibitory microRNA, which is known to bind directly to the 3′UTR region of, and inhibit, myelin regulator factor (MYRF), a transcription factor that is critical for OPC maturation. The overexpression of an miR-615-5p sponge with a precise complementary binding site to miR-615-5p in inflammatory microglia in both EAE and cuprizone-induced models of demyelination was able to ameliorate disease progression and promote remyelination [178]. This suggests that MGEVs from pro-inflammatory microglia may prevent myelination via the miR-615-5p/MYRF axis, identifying miR-615-5p as a potential new therapeutic target in demyelinating conditions. Table 2 highlights the detrimental properties of microglial exosomes reported in recent studies.

## 5. Therapeutic Prospects of Microglial Exosomes in Neurological Injury and Disorders

Despite the pathogenic effects of pro-inflammatory microglia-derived MGEVs, exosomes generated from healthy microglia or engineered with selective cargoes or surface moieties are promising therapeutic agents for neurological disease and injury. The ability of MGEVs to cross the BBB is particularly relevant for them to be vehicles for drug delivery, enabling the noninvasive, systemic administration to specific regions of the brain or the spinal cord that is often a challenge in treating CNS disorders [186,187,188]. Studies have shown that engineered exosomes can effectively be used to deliver a wide range of therapeutic molecules across the BBB, including siRNAs, for targeting key pathological proteins in neurological disorders like AD [189]. Depending upon the phenotypic state of parent microglia, the released MGEVs carry a range of proteins, surface receptors, chaperones, and miRNAs associated with neuroprotective and pro-reparative responses [190] (Figure 3). MGEVs released by pharmacologically or genetically modulated microglia can ameliorate neurodegeneration and promote tissue repair by delivering growth factors to injured neurons and glia, making them valuable for treating conditions involving neuronal damage, such as SCI, TBI, and stroke. The MGEVs’ capacity to facilitate intercellular communication within the CNS by transferring bioactive molecules to other neural cells offers the potential for targeted therapeutic interventions. Leveraging the beneficial aspects of MGEVs while mitigating their harmful effects could therefore pave the way for innovative treatments targeting the CNS.

The therapeutic potential of MGEVs largely depends on engineering strategies that improve their specificity, cargo capacity, and delivery efficiency. These strategies include purifying specific MGEV populations and modifying them for targeted therapeutic use. One of the most promising applications is the delivery of a therapeutic cargo, such as neuroprotective agents, i.e., small interfering RNAs (siRNAs) [15,191], or as new generation vehicles for drug delivery [192], directly targeting specific cellular populations in the CNS. This targeted delivery minimizes off-target effects and enhances therapeutic efficacy.

### 5.1. Monitoring Treatment Response and Disease Progression

MGEVs hold the potential to serve as diagnostic and prognostic biomarkers, showing great promise for the treatment of neurological diseases such as AD, PD, and stroke [193,194,195,196,197,198]. Using MGEV specific markers, exosomes identified in the CNS tissue or peripheral body fluids have been identified to be of microglial origin. For example, in PD, exosomes isolated from the CSF of patients, identified by the microglial marker CD11b, have been found to carry higher levels of oligomeric α-synuclein [25]. Similarly, proteomic and biological profiling discovered that in AD patients, brain-derived MGEVs contain elevated levels of signature proteins such as β-amyloid and tau, along with glial-specific molecules like ANXA5, VGF, GPM6A, and ACTZ. In body fluid samples derived from AD patients, the combination of β-amyloid, tau, and these brain-specific proteins could serve as potential biomarkers [24]. Another study demonstrated that CD11b-positive MGEVs could be obtained from the parietal cortex of cryopreserved human brain tissue of late-stage AD and a comparison with age-matched control cases analyzed using integrated multiomics identified an increase in disease-associated markers like FTH1, TREM2, upregulated levels of tau and synaptic proteins, and elevated cholesterol and reduced DHA-containing lipids as well as specific miRNAs (miR-28-5p, miR-381-3p, miR-651-5p, and miR-188-5p) that are linked to immune and cellular senescence [70].

Recent studies have indicated that MGEVs derived from pro-inflammatory microglia show elevated levels of CD11b^+^/TMEM119^+^/CD14^+^ on their surface [66,194]. Combining these MGEV-specific markers with techniques such as immunoprecipitation and fluorescent nanoparticle tracking, these MGEVs can be detected via liquid biopsy, which will be a future tool for the measurement of microglia activity and the extent of neuroinflammation that accompanies the different neurodegenerative disease courses in various CNS pathologies [199]. Analysis of the dynamic exosome cargo will provide accurate insights into disease progression and treatment response, enabling personalized therapeutic strategies. These longitudinal changes in MGEV biomarkers can reflect the effectiveness of treatments or indicate disease progression, providing real-time feedback for clinical decision-making. Recent studies by Santiago et al. demonstrated state-specific proteomic and transcriptomic signatures of unique proteins and mRNA and microRNA signatures in MGEVs influenced by the activation state of the parent cells [200]. As carriers of diverse biomolecules that mirror the physiological and pathological states of their parent cells, MGEVs are therefore emerging as promising biomarkers for CNS diseases. Continued research efforts are needed to elucidate their role as reliable biomarkers and to overcome technical and clinical challenges for their implementation in routine clinical practice.

### 5.2. Modulation of Exosomal Cargo

Another therapeutic approach focuses on modifying the cargo of MGEVs to enhance their beneficial effects and minimize harmful impacts. By altering the miRNA, protein, or lipid content, exosomes can be tailored to improve therapeutic outcomes. For instance, loading MGEVs with miRNAs that target pro-inflammatory pathways or support neuronal survival can promote recovery, as demonstrated in a study where the administration of miR-17-92 cluster-enriched exosomes derived from human bone marrow mesenchymal stem cells (MSCs), which are known to promote neuronal survival and axonal growth, showed tissue protection, enhanced neuroplasticity, and restored function in rat models of stroke [201] and TBI [202]. Similarly, modulating the parent cells to prevent the incorporation of deleterious proteins or miRNAs, such as toxic tau or α-synuclein, in the MGEVs could help mitigate the progression of neurodegenerative diseases like AD and PD.

Several preclinical studies have found that the polarization of the parent microglial cells from an inflammatory to a neuroprotective or pro-reparative phenotype improves histopathological and functional outcomes in several neurological conditions [203,204]. One example in this regard is that the polarization of parent microglial cells with the anti-inflammatory cytokine IL-4 generated MGEVs that exhibited a reduction in neuronal impairment and mitochondrial dysfunction in rodent models of AD [135]. Similarly, the polarization of parent microglial cells resulted in elevated levels of miR-124-3p in MGEVs, which showed neuroprotective effects by reducing neuroinflammation and contributing to neuronal growth and plasticity after TBI [89] as well as alleviated brain damage and improved the outcome following stroke [203].

Another alternate therapeutic strategy to modulate MGEV cargo would be to involve selective manipulations through the preconditioning of the parent microglia to modulate their cargo to enhance beneficial effects and block pathogenicity. One such approach is preconditioning the parent cells to generate anti-inflammatory MGEVs with therapeutic cargoes. Previous studies with MGEVs derived from hypoxia-preconditioned microglia showed that these exosomes promoted angiogenesis, suppressed apoptosis, and were more effective in promoting neurological recovery after ischemic stroke by activating the TGF-β/Smad2/3 signaling pathway [45]. Similarly, preconditioning macrophages to low-dose endotoxins, such as LPS, resulted in the secretion of MGEVs with an enhanced capacity to confer neuroprotection [205,206] and which improve function after ischemic stroke [207].

### 5.3. Challenges and Considerations

Despite the therapeutic potential of MGEVs, several key challenges remain to be addressed prior to their use for clinical translation as a therapy for neurological pathologies. These challenges involve refining MGEV isolation and purification techniques, overcoming the inherent variability in exosome populations, improving the efficiency of targeted delivery, and ensuring long-term safety and effectiveness across various neurological disorders, which are critical steps for clinical translation [208]. Although different MGEV isolation techniques have been adapted in research laboratories including ultracentrifugation, ultrafiltration, size exclusion chromatography, immunoaffinity methods, polymer precipitation, and microfluidics-based methods, each method is associated with limitations [209,210]. It is crucial to have a standardization of isolation and characterization methods to overcome batch-to-batch variation and achieve consistent yield and purity. Similarly, the validation of biomarker specificity is essential and dependent on the availability of robust exosome analytical platforms prior to use in clinics. Additionally, it is critical to thoroughly characterize exosomes, particularly in terms of their size, morphology, concentration, the presence of exosome-enriched markers, the cargo they carry, and most importantly to confirm the absence of deleterious contaminants.

EVs have distinct features, including stability, low immunogenicity, and high biocompatibility, making them excellent platforms for drug delivery [211]. This enables them to evade removal by the immune system and effectively deliver drugs to target cells. Additional features of EVs, such as their capacity to cross the BBB for CNS drug delivery and their ability to co-deliver multiple drugs within the same exosome while at the same time preserving their pharmacological properties, further enhance their suitability as drug carriers [212]. In addition, the loaded cargo within the EVs is protected from degradation by enzymes due to their lipid bilayer structure, thereby enhancing the stability of the packaged contents [213]. Currently, engineering techniques such as surface modification and efficient cargo loading methods are actively being researched to improve therapeutic efficacy while maintaining exosome integrity. The incorporation of therapeutic molecules into EVs can be performed either through donor cell engineering or by direct loading [213]. Designing engineered EVs with surface-modified peptides that can target specific cell populations is anticipated to be an effective therapeutic approach for CNS pathologies.

MGEVs originating from activated microglia are recognized as carriers of pro-inflammatory cytokines and chemokines, which can activate astrocytes, sustain neuroinflammation, and trigger cell death [46,155]. In various neuropathological conditions, MGEVs have been observed to transfer reactive oxygen species (ROS) and other neurotoxic molecules to surrounding cells, resulting in oxidative stress and neuronal damage [165]. In neurodegenerative diseases such as AD and PD, MGEVs have been implicated in the transport of pathological proteins like amyloid-β and α-synuclein, facilitating their transfer between neurons [69,163,164]. This process contributes to neuronal dysfunction and disease progression [69]. Thus, developing innovative strategies for bioengineering MGEVs to carry therapeutic cargo such as siRNA, miRNA, neurotrophic growth factor proteins, or selective antibodies targeting pathogenic proteins are urgently needed to mitigate harmful effects, modulate disease progression, and enhance treatment outcomes. An example of the use of bioengineered MGEVs has been carried out in AD, where abnormal lysosomal function in microglia leads to the inefficient clearance of Aβ aggregates. Hao et al. (2020) designed mannose-conjugated macrophage-derived exosomes (MExo) loaded with gemfibrozil (Gem) as a microglia-targeting drug delivery system. This approach selectively delivers Gem to microglia, restoring lysosomal activity to enhance Aβ clearance and improve cognitive function in Alzheimer’s disease [115]. To improve cell-selective uptake within the CNS, the surface functionalization of MGEVs with targeted peptides, antibodies, or proteins by conjugating them with specific membrane proteins like LAMP-2B, a member of the lysosome-associated membrane protein (LAMP) family that is found in abundance on the exosome surface, can be employed for the display of a targeting motif. This approach can boost therapeutic efficacy by enhancing interaction in a cell-selective manner while minimizing off-target effects [214,215,216].

Although MGEV research is still evolving, it is essential to recognize the therapeutic potential that is revealed by several studies which have demonstrated that MGEVs carry bioactive molecules capable of modulating neural environments. This opens avenues for therapy, especially in the context of neurotrauma and neurodegenerative diseases.

Moreover, because MGEVs mirror the phenotypic state of their microglial cell origin, there is the potential to develop therapies that enhance the release of protective EVs while reducing neurotoxic ones. Utilizing MGEVs as a therapeutic approach could also modulate key signaling pathways necessary for maintaining microglial balance and homeostasis. However, precisely controlling EV interactions to achieve targeted modulation is challenging, as the nuanced responses of homeostatic microglia to EV signals can lead to unpredictable outcomes, complicating the design of specific therapies. Additionally, MGEVs are only one part of a plethora of responses elicited by microglia, with other effects produced through the direct secretion of molecules (cytokines, trophic factors) and physical interactions such as the phagocytosis of debris, apoptotic cells, or foreign materials, among other activities. While MGEVs may contain several essential molecules, they do not contain the appropriate machinery like the parent cell to respond to external effectors and ligands, which requires specific signaling as well as the direct physical interaction of the microglia with other cell types. Therefore, MGEVs are only one part, albeit an important part, of the repertoire of microglia activities during disease or repair.

It is also important to take into consideration that the effective use of exosomes also lies in achieving targeted delivery, where exosome uptake occurs in a highly cell-selective manner, ensuring only specific cell types internalize the therapeutic cargo. This specificity is crucial to avoid off-target effects and enhance therapeutic efficacy. Although engineering MGEVs for selective targeting remains complex and requires further development, it nevertheless offers significant promise for advancing CNS repair and resilience therapies.

**Figure 5:**
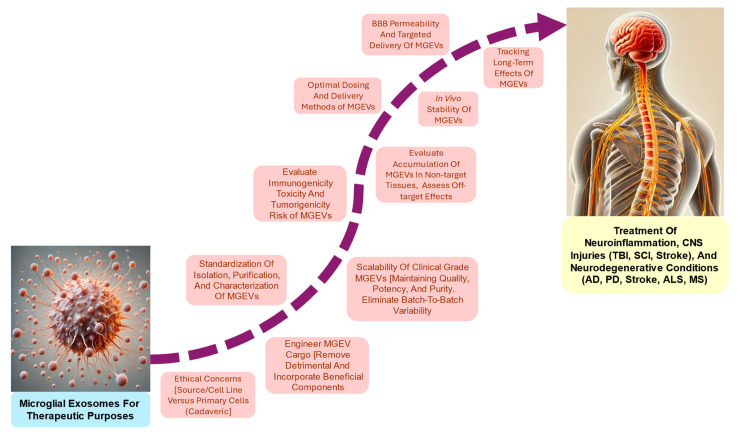
Critical steps for translating microglial exosomes from bench to bedside.

As of now, the FDA (U.S. Food and Drug Administration) has not approved any exosome-based therapies for clinical use. Exosomes from disparate parent cells are under investigation in various preclinical and clinical studies for their potential in treating a range of conditions, including cancer, neurodegenerative diseases, and other disorders. In recent years, exosomes have been explored in preclinical and clinical studies for various neurodegenerative conditions such as AD, PD, and stroke [217]. Several studies in experimental models of CNS injury and disease have demonstrated the therapeutic benefits of Schwann cell (SC)-, mesenchymal stem cell (MSC)-derived, and neural stem cell (NSC)-derived EVs [218,219,220,221,222], with more recent research highlighting the potential of the repeated administration of allogeneic Schwann cell-derived EVs in an FDA-approved single patient study with ALS that did not exhibit any adverse effects towards SCEV administration [223]. However, to date, no clinical trials have been conducted for MGEVs. To successfully translate the preclinical successes of modulated MGEVs into clinical studies, it will be necessary to gain a deeper understanding of consistent therapeutic outcomes, pharmacokinetics, biodistribution, and long-term effects in humans, particularly to address regulatory and safety challenges (Figure 5).

## 6. Conclusions

In conclusion, the therapeutic potential of MGEVs in treating neurological injuries and disorders is promising, yet several challenges must be addressed before clinical application. Advances in engineering techniques are essential to optimize MGEVs for targeted delivery, enhance cargo capacity, and ensure specificity in treating a variety of CNS injuries and diseases. Moreover, the potential of MGEVs as diagnostic and prognostic biomarkers could revolutionize how neurological diseases are monitored and treated, enabling more personalized therapeutic approaches. However, obstacles such as refining isolation and purification methods, improving the consistency of exosome populations, and addressing safety concerns remain significant. Continued research is critical to unlock the full therapeutic promise of MGEVs and overcome the technical and regulatory hurdles for their safe and effective use in clinical practice.

## Figures and Tables

**Table 1:** Beneficial effects associated with microglial exosomes.

Trigger	EV Cargo	Beneficial Effects	Reference
IL4 and TBI	miR-124-3p	Confers neuroprotection/induces microglial polarization and neurite outgrowth by suppressing mTOR signaling via inhibiting PDE4B expression.	Huang et al., 2018 [89]
IL4/Ischemia/ tMCAO	miR-124	Reduces infarct volume; attenuates behavioral deficits by reducing neuronal apoptosis. Targets ubiquitin-specific protease 14 (USP14) to promote cell survival.	Song et al., 2019 [51]
IL4/Cerebral stroke	miR-124	Prevents astrocyte proliferation/promotes the dedifferentiation of astrocytes leading to reduced astrogliosis by targeting STAT3 and Notch1 expression. Increases Sox2 expression in astrocytes. Transforms astrocytes into neuronal progenitor cells.	Li et al., 2021 [92]
IL4/Cerebral ischemia	miR-23a-5p	Reduces brain atrophy volume/promotes white matter repair and functional recovery by targeting Olig3 to increase OPC proliferation, survival, and differentiation.	Li et al., 2022 [149]
IL4/Cerebral ischemia/ MCAO	miR-23a-5p	Attenuates BBB disruption and IgG leakage/decreases brain infarct and edema volume and improves neurological score by targeting TNF and regulating MMP3 and NFκB p65 expression/increasing ZO-1, occludin, and claudin-5 expression.	Pan et al., 2024 [97]
Wild-type hSOD1^WT^ and mutated hSOD1^G93A^/ALS	miR-146a	Confers neuroprotection by increasing SOD1 and reducing pro-inflammatory cytokines.	Vaz et al., 2019 [151]
Intermittent hypoxia	miR-146a-5p	Inhibits mitochondrial reactive oxygen species by targeting HIF1α and regulating neuronal NLRP3 inflammasome and the secretion of inflammatory mediators; alleviates neuronal inflammation.	Zhang et al., 2023 [98]
miR-711 or 1,4,5-trisphosphate 3-kinase B (Itpkb)/ rmTBI	miR-711	Increases ratio of M2/M1 microglia; alleviates neurodegeneration/ improves cognitive function by inhibiting Itpkb-mediated hyperphosphorylation of tau.	Zhang et al., 2020 [152]
IL4/Ischemic brain injury (tMCAO)	miR-135a-5p	Suppresses apoptosis/promotes neuronal survival; reduces brain injury by downregulating the expression of NLRP3 via TXNIP signaling axis.	Liu et al., 2021 [96]
IL4/Ischemic injury	miRNA-137	Attenuates neuronal apoptosis and alleviates brain injury by targeting Notch1.	Zhang et al., 2021 [95]
SCI	miR-151-3p	Suppresses neuronal apoptosis/induces axonal regrowth and promotes functional recovery by downregulating the p53/p21/CDK1 signaling cascade.	Li et al., 2022 [106]
1070 nm light/AD	mmu-miR-7670-3p	Drives microglia towards anti-inflammatory phenotype/suppresses neuroinflammation/alleviates the β-amyloid burden to improve cognitive function by downregulating ATF6 during ER stress/attenuates production of the Aβ peptide/protects dendritic spine integrity.	Chen et al., 2023 [52]
MCAO/R/Oxygen–glucose deprivation/Reoxygenation (OGD/R)	mir-212-5p	Confers neuronal protection and improves functional recovery by targeting PLXNA2.	Li et al., 2023 [153]
Hypoxia preconditioning/Ischemic cerebral stroke	TGF-β1	Induces microglial polarization to an anti-inflammatory phenotype/prevents cell death/promotes angiogenesis/improves functional recovery by activation of the TGF-β/Smad2/3 signaling pathway.	Zhang et al., 2021 [45]
pRS-shTREM2/AD	TREM2	EVs bind to Aβ in a TREM2-dependent manner; expressed on EV membranes and accelerates microglial clearance of amyloid-β.	Huang et al., 2022 [144]

**Table 2:** Detrimental effects associated with microglial EVs.

Inducer or Pathological Condition	EV Cargo	Detrimental Effects	Reference
LPS and Th1 cytokines	miR-146a-5p	Targets expression of presynaptic synaptotagmin1 and postsynaptic neuroligin1; decreases dendritic spine density and excitatory synapses.	Prada et al., 2018 [47]
Glutaminase C/Early AD pathogenesis	miR-146a/miR-130/miR-145a/miR-23b	Induces microglial activation. Downregulates anti-inflammatory miRNAs (miR-124 and let-7b)/promotes a pro-inflammatory microenvironment.	Gao et al., 2019 [143]
LPS/Depression	miR-146a-5p	Decreases neurogenesis and spontaneous discharge of excitatory neurons by targeting Krüppel-like factor 4 (KLF4).	Fan et al., 2022 [55]
Cerebral ischemia/Oxygen–glucose deprivation (OGD)	miR-424-5p	Mediates cerebral endothelial cell injury/damages microvascular membrane permeability by targeting FGF2/STAT3 signaling/induces neurological dysfunctions.	Xie et al., 2020 [179]
Ethanol/ Alcoholism	miRNA let-7b/HMGB complexes	TLR-7-associated neuroimmune gene induction; induces neurodegeneration.	Coleman et al., 2017 [180]
LPS/Neovascular retinal diseases	miR-155-5p	Promotes angiogenesis by targeting Irf1/miR-155-5p/Socs1 signaling axis.	Chen et al., 2023 [56]
EAE/Cuprizone (CPZ)-induced demyelination	miR-615-5p	Inhibits OPC maturation and remyelination by targeting myelin regulator factor (MYRF).	Ji et al., 2024 [178]
ATP	Endocannabinoids, N-arachidonoylethanolamine (AEA)	Activates type 1 cannabinoid receptors (CB1) and inhibits presynaptic transmission in target GABAergic neurons, contributing to alteration of excitation/inhibition balance.	Gabrielli et al., 2015 [140]
LPS/ATP/Tau	Tau	Increases tau propagation in recipient neurons.	Asai et al., 2015 [28]
LPS/ATP/Tau	Tau	Promotes tau propagation in healthy neurons.	Clayton et al., 2021 [117]
Monomeric and oligomeric α-syn/PD	a-synuclein	Enhances microglia proliferation and arborization. Dysregulates autophagy in parent microglia from phagocytosed α-synuclein, causing accelerated secretion and aggregate formation of α-synuclein in the recipient neurons; exacerbates disease propagation.	Xia et al., 2019 [114]
Preformed fibril (PFF)/LPS/PD	α-synuclein/ Pro-inflammatory cytokines	α-synuclein-mediated protein aggregation in recipient dopaminergic neurons; propagates degeneration/loss of dopaminergic neurons.	Guo et al., 2020 [25]
Aβ42/AD	Misfolded Aβ proteins	Transfers misfolded Aβ proteins to healthy neurons in a transsynaptic manner/alters dendritic spine morphology/ propagates synaptic dysfunction.	Gabrielli et al., 2022 [169]
Th1 cytokines/Lysolecithin-induced myelin lesions	IL-1a, C1q, IL-1β, iNOS, SOCS3	Blockade of OPC differentiation and maturation to prevent remyelination.	Lombardi et al., 2019 [145]
Ethanol/Fetal alcohol spectrum disorders (FASDs)	Complement protein C1q, CD13, MMP2	Complement C1q-mediated ROS production and cellular death of β-endorphin neurons.	Mukherjee et al., 2020 [175]
OGD/Transient MCAO	PDE1B	Upregulation of autophagic flux and exosome release, inducing neuronal death.	Zang et al., 2020 [181]
Focal cerebral ischemia/Glutaminase (GLS)	Glutaminase	Induction of GLS-induced oxidative stress and excitotoxicity; induces neurotoxicity.	Gao et al., 2020 [182]
Focal cerebral ischemia/GLS	Glutaminase	Induction of GLS-induced oxidative stress and excitotoxicity/triggers neuroinflammation and induces neurotoxic microenvironment.	Ding et al., 2021 [183]
Divalent manganese/LPS	Apoptosis-associated speck-like protein containing a caspase recruitment domain (ASC)	Activates NLRP3 inflammasome to promote neuroinflammation and stimulate neurotoxicity.	Sarkar et al., 2019 [184]
α-synuclein/PD	Cathepsin L	Induces neurotoxicity via P2X7R/PI3K/AKT signaling.	Jiang et al., 2022 [185]

## Data Availability

Not applicable.
